# Effects of race and sex on cerebral hemodynamics, oxygen delivery and blood flow distribution in response to high altitude

**DOI:** 10.1038/srep30500

**Published:** 2016-08-09

**Authors:** Jie Liu, Yang Liu, Li-hua Ren, Li Li, Zhen Wang, Shan-shan Liu, Su-zhi Li, Tie-sheng Cao

**Affiliations:** 1 Department of Ultrasound Diagnostics, Tangdu Hospital, Fourth Military Medical University, Xi’an, Shaanxi, China; 2 Department of Cardiovascular Surgery, Xijing Hospital, Fourth Military Medical University, Xi’an, Shaanxi, China; 3 General Hospital of Tibet Military Area Command, Lhasa, Tibet Autonomous Region, China; 4 Department of Ultrasonic Medicine, Affiliated Hospital of Tibet University for Nationalities, Xianyang, Shaanxi, China

## Abstract

To assess racial, sexual, and regional differences in cerebral hemodynamic response to high altitude (HA, 3658 m). We performed cross-sectional comparisons on total cerebral blood flow (TCBF = sum of bilateral internal carotid and vertebral arterial blood flows = Q_ICA_ + Q_VA_), total cerebrovascular resistance (TCVR), total cerebral oxygen delivery (TCOD) and Q_VA_/TCBF (%), among six groups of young healthy subjects: Tibetans (2-year staying) and Han (Han Chinese) at sea level, Han (2-day, 1-year and 5-year) and Tibetans at HA. Bilateral ICA and VA diameters and flow velocities were derived from duplex ultrasonography; and simultaneous measurements of arterial pressure, oxygen saturation, and hemoglobin concentration were conducted. Neither acute (2-day) nor chronic (>1 year) responses showed sex differences in Han, except that women showed lower TCOD compared with men. Tibetans and Han exhibited different chronic responses (percentage alteration relative to the sea-level counterpart value) in TCBF (−17% *vs*. 0%), TCVR (22% *vs*. 12%), TCOD (0% *vs*. 10%) and Q_VA_/TCBF (0% *vs*. 2.4%, absolute increase), with lower resting TCOD found in SL- and HA-Tibetans. Our findings indicate racial but not sex differences in cerebral hemodynamic adaptations to HA, with Tibetans (but not Han) demonstrating an altitude-related change of CBF distribution.

As the human activity at high altitude (HA) has increased, common healthcare problems that are related to hypobaric hypoxia such as acute and chronic mountain sickness (AMS and CMS)^1^,^2^ have drawn more attention. These diseases appear more often in immigrant lowlanders, with various incidence and hypoxic tolerance patterns observed among different native highlanders (such as Tibetans, Andeans and Ethiopians)^3^. Thus race (or genetic) factors may have a significant impact on the physiological adaptations to the sustained hypoxia^4^.

Some studies have demonstrated three typical racial patterns of HA adaptation in hematological and cardiopulmonary responses^3^, i.e. Andean (erythrocytosis with arterial hypoxemia), Tibetan (normal hemoglobin concentration with arterial hypoxemia), and Ethiopian (normal hemoglobin concentration and arterial oxygen saturation) patterns. However, limited knowledge is available about the racial differences in hemodynamic responses to HA in the brain^5^,^6^, which is the most oxygen dependent organ. In this regard, few studies have been performed in Tibetans (i.e. Himalayans or Sherpas) for evaluating brain perfusion by cerebral blood flow velocity (CBFV) at the middle cerebral artery^7^ or internal carotid artery (ICA)^8^ using transcranial Doppler (TCD), which have been limited by no available measurement on real volumetric cerebral blood flow (CBF, or abbreviated as Q). One study found that Tibetans exhibited similar ICA CBFV, diameter and cerebral oxygen delivery as Han Chinese (or abbreviated as Han) at the altitude of 3658 m; however, the study had a small sample size with only male subjects and they showed no racial difference in hemoglobin concentration (Hb)^9^, which suggests that further confirmation with improved study design and technique may be needed.

There are limited data available regarding long-term (>1 year) HA exposure when compared with short-term (few hours to few months) exposure and none of them have explored sex differences^10^. In addition, the cerebral hemodynamic response to hypoxia may differ between anterior and posterior circulations^11^,^12^,^13^,^14^ with the correspondingly altered CBF distribution, despite the presence of the circle of Willis and other downstream collateral circulations^15^. Filling this gap in the literature may also help to determine an adaptation pattern that may be more beneficial or less detrimental to brain structure^16^ and function^17^ when exposed to hypobaric or clinical hypoxia.

The aim of this cross-sectional study was to examine the effects of short- and long-term HA exposures on cerebral hemodynamics using advanced duplex ultrasonography (Fig. 1) between female and male, Tibetan (major native highlanders) and Han (lowlanders) in China when compared with a similar population staying at sea level (SL). In particular, we sought to determine whether resting cerebral hemodynamic values and CBF distribution have racial or sexual differences, and whether the cerebral hemodynamic responses to HA exposure would have racial, sexual, or regional (i.e. anterior *vs*. posterior) differences. We hypothesized that highlander Tibetans would possess a distinct cerebral hemodynamic phenotype as a reflection of better HA adaptation than lowlander Hans.

## Results

All groups were similar in demographic features except that the Han-HA-5 yr group was ~2–4 years older than the other groups (Table 1), which was expected because the Han-HA-5yr included veteran soldiers while the other HA groups were new soldiers.

### Impact of short- and long-term HA exposures on female and male Han Chinese

Blood pressure was increased and SaO_2_ was decreased in all Han-HA groups relative to the SL counterpart group (i.e. Han-SL), with the Han-HA-2d showing the greatest changes (Table 1). Heart rate was also significantly increased in Han-HA-2d but returned close to the Han-SL level in Han-HA-1yr and Han-HA-5yr. The hematocrit (Hct) showed a slight increase in Han-HA-2d with a greater increase in Han-HA-1yr and Han-HA-5yr, when compared with Han-SL. By contrast, the Hb was not higher in the Han-HA-2d when compared with Han-SL, but a significant increase in Han-HA-1yr (16% in both females and males relative to their SL counterpart values) and Han-HA-5yr (22% and 18% in females and males) was observed (*P* < 0.001). As a result, the CaO_2_ decreased (−9% and −10% in females and males) in Han-HA-2d but increased in Han-HA-1yr (9% in both) and Han-HA-5yr (15% and 11% in females and males), when compared with the Han-SL (*P* < 0.001).

All Han HA groups showed wider ICA and VA diameters, with no differences or even a slight decrease in their CBFVs, compared to the Han-SL; most of the derived regional CBFs showed a trend of increase in Han-HA-2d, but return to the SL level in Han-HA-1yr and Han-HA-5yr. When analyzed as a global bilateral measure, VA appeared to have a slightly greater increase in CBF than ICA (17% *vs*. 15%, relative to the SL counterpart values) under short-term HA exposure (*P* = 0.02 indicated by the 2-way ANOVA; Fig. 2A); however, the comparisons on Q_VA_/TCBF among different altitude groups showed no significant differences (Table 1 and Fig. 2C).

The change of TCBF was consistent with that of regional CBF with a significant increase (16% in both females and males) under acute (2-day) HA exposure and a return to the SL level under chronic (1- or 5-year) HA exposure (*P* < 0.01). In contrast, the TCVR did not change under 2-day HA exposure, but tended to increase after 1-year HA exposure (8% and 9% in females and males, *P* = 0.17 and 0.08) and significantly increased after 5-year HA exposure (17% and 15% in females and males, *P* = 0.01 and 0.02), when compared with Han-SL. Similarly, the TCOD did not change with 2-day HA exposure, but tended to increase after 1-year exposure (8% in both females and males, *P* = 0.13 and 0.07) and significantly increased after 5-year exposure (14% and 11% in females and males, *P* = 0.01 and 0.04), when compared with Han-SL.

Although there were significant sex differences in resting values at both SL and HA for some parameters (Table 1), both sexes appeared to respond similarly to HA, as confirmed by the lack of a significant Altitude × Sex interaction from 2 –Way ANOVA comparisons.

### Comparisons between Tibetans and Han Chinese under long-term SL and HA exposures

Tibetans (*vs*. Han) showed higher SBP, lower Hct, Hb, and CaO_2_ at SL; but appeared to have lower blood pressures, Hct, Hb, and CaO_2_ at HA (Table 2). Both Tibetan-HA and Han-HA groups showed significantly decreased SaO_2_ (−6% relative to their SL counterpart values) accompanied with increased Hct (21% and 15%), Hb (29% and 19%) and CaO_2_ (22% and 12%) when compared with the SL groups. In contrast, blood pressure and heart rate was not significantly different between SL and HA Tibetans, but increased significantly (8~12%) in Han at HA than at SL. The 3-way ANOVA demonstrated a significant Altitude × Race interaction (reflecting race-related differences to HA exposure) for blood pressure (SBP, DBP and MAP; *P* < 0.01), heart rate (*P* = 0.03), Hb (*P* = 0.02) and CaO_2_ (*P* < 0.01), with no significant sex interactions (i.e. Altitude × Sex or Race × Sex; Table 3).

The racial differences in cerebral large vessel parameters were mainly found in the ICA but not in the VA at both SL and HA. Tibetan and Han HA groups demonstrated consistent decreases in all regional CBFVs, but with only Han group displaying appreciable increases (9~11%) in all vessel diameters at HA relative to the SL counterpart values (*P* < 0.05). All the regional CBFs at HA remained constant in Han but tended to decrease in Tibetans, when compared to their SL counterparts. The 3-way ANOVA demonstrated a significant Altitude × Race interaction on bilateral ICA diameters and CBFs (*P* < 0.01), with no significant sex interactions. When further analyzing regional differences between anterior (sum of bilateral ICA) and posterior (sum of bilateral VA) CBF responses to long-term HA exposure in Tibetans, the posterior circulation appeared to have a lower decrease in CBF than the anterior circulation (−11% *vs*. −19%, relative to the Tibetan-SL; *P* < 0.001; 2-way ANOVA; Fig. 2B). Moreover, Q_VA_/TCBF showed a significant altitude-related increase in Tibetan-HA compared with Han-HA (Fig. 2D, especially for males as shown in Table 2).

Resting TCBF [mL/min] showed race differences at SL (with a trend of higher levels in Tibetans than in Han, *P* = 0.06 for pooled female and male data) and HA (−12% lower in both Tibetan females and males, *P* < 0.01), with no sex differences. The TCBF appeared to remain constant in Han from SL to HA but significantly decreased in Tibetans (−17% in females, and −18% in males, *P* < 0.01) after long-term HA exposure. TCVR was significantly increased at HA relative to the SL counterpart value, in both Han (12% in females, and 11% in males, *P* = 0.03) and Tibetans (22% in both females and males, *P* < 0.01). The resting TCOD [ml O_2_/min] showed both race and sex differences (lower in Tibetans and females) at either SL or HA, but kept unchanged in Tibetans and significantly increased in Han (11% in females, and 9% in males, *P* = 0.03) at HA, with comparison to the SL counterpart value (Table 2 and Fig. 3). The 3-way ANOVA (Table 3) confirmed that the sex did not interact with race or altitude to impact on the major cerebral parameters, with only race interacting with altitude (Altitude × Race) to affect TCBF (*P* < 0.001), TCVR (with a trend of *P* = 0.09), TCOD (*P* = 0.02) and Q_VA_/TCBF (*P* = 0.04).

## Discussion

To the best of our knowledge, this is the first study to assess racial, sexual and regional differences of cerebral hemodynamic responses to altitude change in Chinese population including Tibetans (native highlanders) and Han (native lowlanders). The key findings from this study were: (1) Han females always appeared to have lower TCOD but similar TCBF and TCVR, compared to Han males. However no sex difference was found in the altitude response of these parameters. (2) When compared with Han, Tibetans appeared to have lower resting TCBF and TCOD, but similar TCVR. Moreover, different racial response to long-term HA exposure was found in TCBF (decreased in Tibetans but maintained in Han), TCVR (more increased in Tibetans than in Han, with a trend of *P* = 0.09) and TCOD (maintained in Tibetans but increased in Han). (3) Tibetans (but not Han) showed an altitude-related change of CBF distribution, as reflected by a less relative decrease of CBF in VA than in ICA after long-term HA exposure, with an increased posterior CBF distribution at HA relative to the SL counterpart value. Collectively, our findings indicate that Tibetans possess a unique pattern of cerebral hemodynamic adaptation to HA compared with Han, with no sexual but regional differences.

The observed acute and chronic altitude responses of systemic physiological parameters in Han are essentially consistent with previous findings from longitudinal studies^18^,^19^,^20^. The analysis of race differences indicate that the immigrant Tibetan residents at SL would keep their blood pressure and heart rate at their HA levels, also not different from those of Han natives at SL; while the immigrant Han residents at HA experienced with a dramatically elevated blood pressure (also higher than those of Tibetan natives at HA) and a trend of increased heart rate. The blood pressure and heart rate responses to HA might be due to a sympathetic nervous system activation^20^ in Han, but appeared to be blunted with nearly no responses in Tibetans. The relative hypertension in Han at HA appears to increase the cardiovascular risk^21^, and the higher resting heart rate might be associated with a reduced reserve for cardiac function^8^. The hematological HA response of Han in the present study also agrees with previous findings on lowlanders^22^, with Hb unchanged on short-term HA exposure but increased on long-term exposure; however, the Hct began to increase even during the first several days at HA, which was likely due to the hypohydration on acute HA exposure^23^. The observed racial difference in hematological parameters at HA between Tibetans and Han^9^ were similar to that between Tibetans and Andeans in previous reports^24^, with consistent result showing that Tibetans at HA exhibited the Hct value within the range expected from lowlanders at SL^25^. Whether the lower erythropoietic response to HA in Tibetans would facilitate a better reserve of oxygen transport (with lower blood viscosity) not only in the muscle^26^ but also in the brain^27^, is yet to be determined. Another interesting finding is that the Tibetans who descended to SL appeared to have lower Hb or Hct, as compared to Han. This feature is consistent with a recent study on Tibetans living at the United Kingdom^28^. It may also represent an racial trait which is independent of the life-long HA exposure, as indicated from the observed lack of difference in Hb between HA-raised and SL-raised Han^16^,^17^.

The obvious dilation of cerebral large-vessels on short-term HA exposure in Han is in line with several recent studies^13^,^18^,^19^,^29^,^30^; although this dilation appeared to be weaker with long-term exposure, the diameter was still above the level at SL^21^. We found the diameter response to HA might have race difference despite no sex difference, i.e. the Tibetans appeared to blunt the chronic diameter response to HA compared with Han, perhaps due to a less passive dilation by a lower blood pressure, or a less vasculature wall shear stress (mediated by a decreased flow in the pial vessels), or a combination of both. Note that this observed large-vessel dilation during the HA-induced acute or chronic hypertension in Han, agrees with the ICA or VA constriction during the acute hypotension induced by lower-body negative pressure^31^, but contradicts the ICA or VA constriction/dilatation during the drug-induced acute hypertension/hypotension^32^, suggesting they may have different underlying mechanisms and that circulating factors may also play a role in. Note that the vessel diameter response should be highly considered to make an accurate and reliable assessment of CBF response to HA since our observed relative change or difference in CBF was actually characterized by the relative change or difference in large-vessel diameter (but not CBFV). Actually the CBFV had no significant altitude or race related difference in the present study, which is consistent with some previous CBFV studies at MCA^30^,^33^, ICA or VA^13^ for acute HA response, and at ICA for race comparison^8^; and sometimes it even behaved in the opposite way for diameter, i.e. decreased with increased diameter. Furthermore, the vessel CBF is proportional to the square of radius (i.e. the vessel cross-sectional area) assuming no change in CBFV, so if we used the CBFV as a surrogate to CBF, it would lead to a substantial error, e.g. in our case the CBFV even decreased rather than increased on HA exposure at some vessels for acute response. Thus, the indicated dilation of cerebral large vessel at such level of hypoxemia supports the idea that some previous studies using TCD at HA might have underestimated the real volumetric CBF^6^,^14^, as also reflected by the increasing concerns on the limitations of TCD (with neither vessel diameter measurement nor accurate angle correction for CBFV measurement)^15^,^30^,^32^,^34^.

Our observed acute TCBF response to HA in Han is consistent with previous longitudinal studies^1^,^35^. This abruptly increased TCBF was accompanied by unaltered TCVR and TCOD in the present study, with the former one suggesting no significant remodeling in cerebral small or resistance vessel on short-term HA exposure^36^, and the latter one indicating that the sudden decrease in SaO_2_ or CaO_2_ was offset by the increased TCBF to maintain the TCOD no less than the SL level^22^,^29^,^30^, which may help to maintain the cerebral metabolism during the acute ascent to HA^37^. While for long-term HA adaptation in Han, the TCBF appeared to return to the SL counterpart level, which agrees with previous studies showing that TCBF returned to baseline levels after acclimatization to HA^27^. Our novel findings are the trends of increasing TCVR and TCOD on longer (>1 year) HA exposure, indicating a potential remodeling of cerebral vasculature and an improved cerebral oxygen supply.

Our study indicates a significantly lower resting TCBF of Tibetans at HA, when compared to the values of the HA lowlanders (Han) and the SL counterpart, respectively. Contrary to our finding, one recent review paper^6^ inferred that Tibetans CBF was slightly higher than the standard SL value^8^. We suspect that its conclusion might be biased by the small sample size, only CBFV measurement, and pooled data from different observers or research groups. We further found the TCOD at HA was well regulated at a constant level (or even slightly upregulated in Han, relative to the SL counterpart value) despite marked changes in altitude and CaO_2_. which is in line with previous findings^22^. This might be due to the coupling between brain perfusion and oxygen metabolism. The observed race differences in the resting TCBF, TCVR or TCOD, and their responses to altitude, support that Tibetans must engage other mechanisms to sustain normal oxygen supply and aerobic metabolism in brain^24^; however, the exact mechanisms, biological significance and clinical implication are still unclear. Whether they are related to an increased arterial-venous oxygen content difference (enhanced cerebral oxygen extraction)^6^, increased non-oxidative metabolism^38^, altered oxygen consumption (improved utilization or less need of oxygen to maintain normal brain tissue metabolism)^39^, inherent different vascular reactivity to O_2_ or CO_2_^7^,^37^, or a combination of all in Tibetans as compared to Han warrants further investigations. Furthermore, whether the lower TCBF and TCOD in Tibetans at HA would facilitate a better reserve on cerebral oxygen transport^27^, during exercise^8^ or cognitive task (brain functional activation), requires to be further studied.

In the present study, women showed similar TCBF and TCVR compared with men but lower TCOD (e.g. −14% lower in Han-SL women than men), which is consistent with previous findings of higher brain perfusion rate (i.e. TCBF/ total brain-tissue volume or weight) in young women (12~13%)^40^,^41^ and similar cerebral metabolism^37^, after adjusting for total brain-tissue volume, which is 13% lower in women^42^. Thus the sex differences in the resting global cerebral hemodynamic values can be explained by the differences in brain-tissue volumes. No sex difference in altitude responses in the present observation seems to contradict with previously reported sex effects on vasomotor reactivity to CO_2_ (higher in women)^43^, carbohydrate metabolism in response to a metabolic stress (altitude, exercise or diabetes), which are attenuated in women^44^, which merits further studies to provide plausible explanations.

Some other interesting results were found in the regional differences of CBF response to HA exposure. We found the increase of CBF on short-term HA exposure in Han appeared to be a little greater in posterior (VA or vertebrobasilar territory) than in anterior (ICA territory) cerebral circulation; however, since the CBF distribution did not show a corresponding difference, and the former observed difference was very slight (~2%, Fig. 2A), we conservatively accept a negative finding on this aspect in Han. Whereas a more convincing result was found in Tibetans on long-term HA exposure, which showed a reduction of a greater magnitude in CBF (~8%, Fig. 2B) in posterior than in anterior circulation relative to the SL counterpart CBF, accompanied by a greater posterior CBF distribution at HA than that at SL. This finding reflects another feature of racial differences in cerebral hemodynamic adaptation to HA. Recently, cerebral hemodynamic differences between anterior and posterior circulations^11^,^12^,^13^,^14^,^15^,^18^,^19^,^34^, have garnered interest because of its clinical relevance to the posterior circulation ischemia. Based on the previous and current findings on Han lowlanders, a preferential maintenance of CBF in posterior circulation is likely to appear during a rapid (<~6 hours) ascent^19^ (or a sudden hypoxia)^11^,^12^,^13^ rather than during a slow (>~12 hours) ascent^18^ (or a HA sojourn and chronic hypoxia) to HA. The favored posterior CBF distribution during transient hypoxia was suggested to be advantageous for protecting regions of the brain with important and necessary homeostatic function, such as the respiratory and cardiovascular control centers (e.g., cerebellum, hypothalamus, thalamus and brainstem)^13^. However, the significant impact of altitude on Tibetans CBF distribution even under long-term exposure was another interesting finding that might have different underlying mechanisms, but may also reflect a favored maintenance of posterior CBF with potential benefit to improve the HA adaptation.

### Strengths and limitations

**Figure 1:**
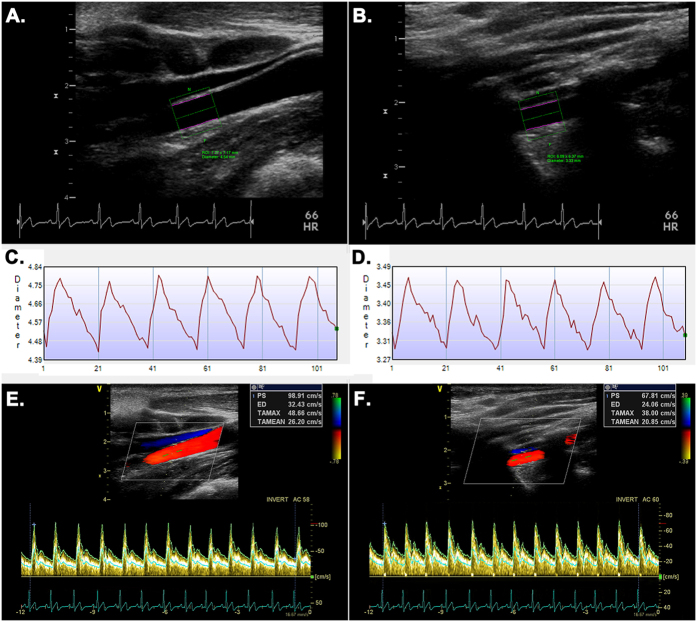
Measurement of cerebral blood flow at internal carotid (ICA) and vertebral arteries (VA) with color-coded duplex ultrasonography. The double purple lines within the region of interest (ROI, green rectangle) are the detected vessel inner walls in the high-resolution video clip of the ICA (**A**) or VA (**B**), which are used to track continuous pulsatile change of vessel diameter at the ICA (**C**) or VA (**D**) using an edge-detection and wall-tracking software (Brachial Analyzer, Medical Imaging Applications LLC, USA). Beat-by-beat recording of blood flow velocity at the site of vessel diameter measurement for the ICA (**E**) or VA (**F**) is presented. PSV, peak systolic velocity; ED, end-diastolic velocity; TAMAX, time-averaged max velocity; TAMEAN, time-averaged mean velocity.

An important strength of this study is the use of non-invasive color-coded duplex ultrasonography to measure CBF (Fig. 1). This technique has the advantage of combining both vessel diameter and CBFV measurements to quantify the volumetric CBF at the major cerebral feeding arteries, which was shown good reproducibility^45^ and high agreement with other validated methods^46^. Another strength is that our subjects were all young and healthy with very similar backgrounds and demographic features, which minimized potential confounders and allowed cross-sectional comparisons.

A limitation of this study was the lack of measurements of end tidal CO_2_ or partial pressure of arterial carbon dioxide (PaCO_2_) (which would affect CBF), and internal jugular venous oxygen saturation (which could be used to estimate the total cerebral metabolic rate of oxygen on the basis of Fick’s law^27^). However, most of previous studies reported no race difference between Tibetans and Han in either resting ETCO_2_^7^,^8^,^47^ or transcutaneous PaCO_2_^48^ at HA, with one study showing a lower ETCO_2_ in Tibetans at SL compared to Han^28^, both of which increase confidence in our results regarding racial differences in resting TCBF and the TCBF response to HA. Another limitation was that total brain-tissue volume was not measured to further assess the total brain perfusion rate. We lacked a Tibetan 2-day HA comparison group in this study, which limited our ability to examine racial differences in acute cerebral hemodynamic response to HA. Finally, the cross-sectional nature of the study should be acknowledged. Future longitudinal or experimental studies could offer confirmatory evidence on the effects of race and sex on cerebral hemodynamic adaptations to short and long-term HA exposures.

### Summary

Taken together, our findings highlight the integrative nature of cerebral hemodynamic response to HA that preserve (or upregulate) oxygen delivery to the brain with no sex difference but racial and conditionally regional differences. We further demonstrated that Tibetans had a lower resting TCOD and different chronic HA response in TCBF, TCVR, TCOD and CBF distribution, as compared to Han, which confirms race differences in adaptive strategy against hypoxic stress on cerebral hemodynamics, suggesting a genetic role in modifying the cerebral hemodynamic phenotype under HA or hypoxia exposure. Note that by our measure, Tibetans (*vs*. Han) appeared to be better adapted to HA life with moderate Hct or Hb, and lower TCBF; however, they might be worse adapted to SL with lower Hct or Hb, and a trend of higher TCBF. Further research in this area is encouraged to yield more important insights into human tolerance and adaptation to acute and chronic states of hypoxia.

## Materials and Methods

### Ethical approval

This study conformed to the standards of the *Declaration of Helsinki* for medical research involving human subjects. All subjects signed the informed consent and the study protocol was approved by the clinical research ethical review board at the Fourth Military Medical University.

### Participants

A total of 121 Tibetans (63 women) and 191 Han Chinese (86 women) volunteered to participate in the study. The Tibetan subjects were born and raised at the Qinghai-Tibetan Plateau (3240~4507 m) for at least 18 years; while the Han subjects were born and raised close to SL (≤400 m) for at least 18 years. None of the subjects had recently (<1 year) travelled to other altitude. Subjects were divided into 6 groups according to the duration of stay at sea level or high altitude: ① Tibetan migrants staying at SL for 2 years (Tibetan-SL-2 yr); ② Tibetan natives at HA (Tibetan-HA); ③ Han natives at SL (Han-SL); ④ Han newcomers at HA for only 2 days (Han-HA-2d); ⑤ Han migrants at HA for 1 year (Han-HA-1yr); and ⑥ Han migrants at HA for 5 years (Han-HA-5yr). All the data were collected at subject’s current altitude, i.e. 400 m (Xianyang and Xi’an) as SL, or 3658 m (Lhasa) as HA.

All subjects were either young college students or army soldiers and were healthy as screened by medical history questionnaire and standard physical examination, which are national requirements for college entry or army enlistment. None of the subjects had any known cerebrovascular, cardiovascular, or pulmonary disorders, nor did they have severe AMS, high-altitude cerebral edema, high-altitude pulmonary edema, or CMS. Subjects were right-handed non-smokers who were not taking any current medication. Subjects refrained from high intensity exercise, alcohol, tea or caffeinated drink for at least 24 hours, and fasted for at least 2 hours after a light meal, before any of the following examinations, which were carried out in a temperature-controlled (23 °C) room within a single day. Physiological data were collected with the subject in a supine position after a blood draw and a 10-minute rest. During data collection, subjects were instructed to keep awake but to not speak or engage cognitively (e.g. reading, thinking, watching television, etc.). All of these procedures helped to allow the stabilization of systemic and cerebral hemodynamics.

### Data collection and measurement

A color-coded duplex ultrasonography system (Vivid E9, GE Vingmed Ultrasound AS, Horten, Norway) with a 3–10 MHz linear array probe was used to measure CBF. The CBF measurement at ICA was performed at least 1 cm away from the carotid bulb; while the CBF at vertebral artery (VA) was measured between the C_4_ and C_6_ intertransverse segments (Fig. 1)^45^. A straight vessel segment (>5 mm) with a parallel-wall view was identified as the site to measure CBF, while the probe was carefully adjusted to obtain the largest longitudinal lumen area at this site, which would help to identify the correct section to measure vessel CBFV and diameter. The Doppler sample volume was positioned at this site to cover the entire lumen to measure CBFV with the insonation angle adjusted to ≤60°. The CBFV in the present paper was specifically referred to as the space- and time-averaged mean velocity (TAMEAN) across the whole vessel lumen (assuming a laminar flow) from at least 6 consecutive cardiac cycles. The vertical distance between the parallel inner walls was determined as the vessel diameter, with its pulsatile change recorded continuously for 6 cardiac cycles on a high-resolution B-mode video clip. Change of the vessel diameter at the sampling segment (with a length of 5~10 mm) was measured off-line using an edge-detection and wall-tracking technology to obtain the time-averaged mean vessel diameter from 3~6 consecutive cardiac cycles (Brachial Analyzer, Medical Imaging Applications LLC, USA) (Fig. 1). The CBFV and diameter measurements were repeated 3 times for each vessel with the averaged values used for CBF calculation. These procedures were taken to reduce the intrinsic CBF variability associated with the respiratory and other low frequency oscillations^49^. The vessel CBF was calculated as the product of CBFV and the vessel mean cross-sectional area (A) as: CBF = CBFV × A × 60 = CBFV × [(mean diameter/2)^2^ × 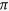] × 60. All the ultrasound data were collected and measured by the same experienced radiologist (JL), who was blinded to other data. Our strict protocol helped to improve the reproducibility of TCBF measurement, with a low intra-observer coefficient of variation ~5% as demonstrated previously^45^.

During the ultrasound examination, brachial systolic and diastolic blood pressure (SBP and DBP) and heart rate were measured with an interval of ~5 minutes between measurements using an electronic sphygmomanometer (HEM-7051, Omron Healthcare, Japan) and arterial blood oxygen saturation (SaO_2_) was measured simultaneously using a finger pulse oximeter (CMS50E, Contec Medical Systems, China). All the above parameters were measured 3 times and averaged values were used for final data analysis.

Venous blood sample was drawn to assess the Hct and Hb. The total CBF (TCBF) was obtained as a sum of bilateral ICA and VA CBFs, i.e. TCBF (mL/min) = Q_ICA_ + Q_VA_. Total cerebrovascular resistance (TCVR) was calculated as: TCVR (mmHg∙min/mL) = MAP/TCBF. The arterial content of oxygen (CaO_2_) was calculated as: CaO_2_ (ml O_2_/dL) = 1.36 (ml O_2_ / g Hb) × Hb (g/dL) × SaO_2_ (%)/100, with the small quantity of dissolved oxygen (decreases further with rising altitude) not included in the estimation^22^. The total cerebral oxygen delivery (TCOD) was calculated as: TCOD (mL O_2_/min) = TCBF (mL/min) × CaO_2_ (mL O_2_/dL)/100. The posterior CBF distribution was assessed by the percentage of posterior CBF (bilateral VA CBFs) to TCBF, i.e. Q_VA_/TCBF (%).

### Data analysis

**Figure 2:**
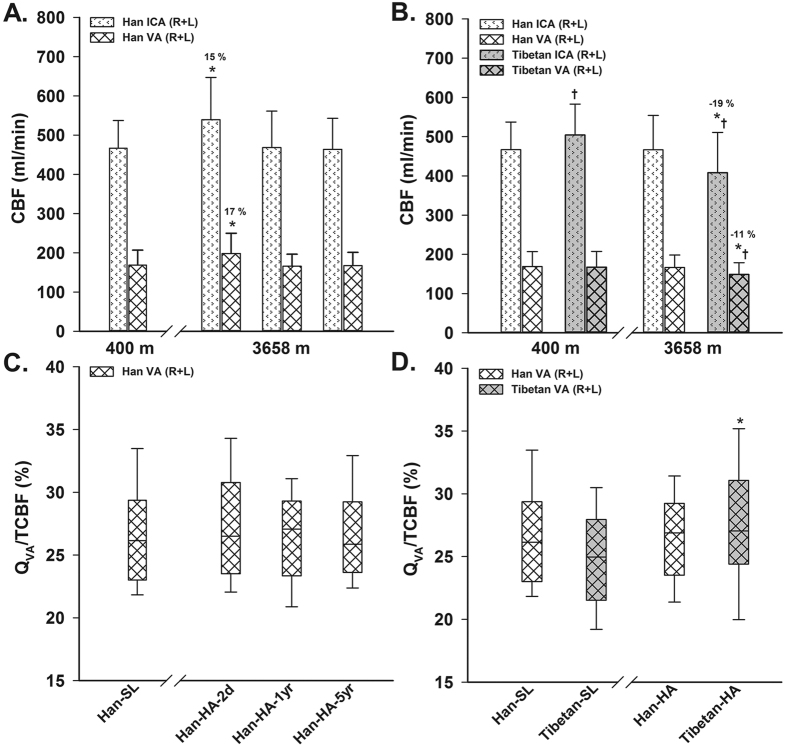
Comparisons of regional cerebral blood flow (CBF) and its distribution on short- and long-term HA exposures in Han Chinese (**A**,**C**), and the race difference on long-term exposure (**B,D**). ICA = internal carotid artery; VA = vertebral artery; R + L = right and left sides = bilateral sides; TCBF = total CBF; Q_VA_/TCBF = the percentage of bilateral VA CBFs to TCBF; SL = sea level (400 m); HA = high altitude (3658 m); Han-SL = Han natives staying at SL; Han-HA-2d = Han newcomers at HA for only 2 days; Han-HA-1yr = Han migrants at HA for 1 year; Han-HA-5yr = Han migrants at HA for 5 years; Tibetan-SL = Tibetan migrants at SL for 2 years; Han-HA = Han migrants at HA for 1 or 5 years; Tibetan-HA = Tibetan natives at HA. Values are mean ± SD. *P < 0.05 *vs*. the SL counterpart, % (in **A,B**) = percentage change of the mean value relative to the SL counterpart; ^†^*P* < 0.05, Tibetans *vs*. Han Chinese at same altitude.

**Figure 3:**
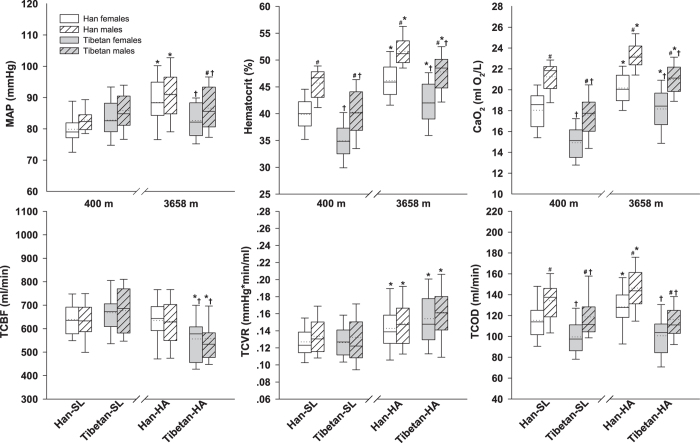
Race and sex differences in major hemodynamic parameters on long-term (>1 year) altitude exposure. Box plots of the mean arterial pressure (MAP), hematocrit, arterial content of oxygen (CaO_2_), total cerebral blood flow (TCBF), total cerebrovascular resistance (TCVR) and total cerebral oxygen delivery (TCOD) in young healthy Han and Tibetan residents at sea level (SL, 400 m) and high altitude (HA, 3658 m). Han-SL = Han natives staying at SL; Tibetan-SL = Tibetan migrants at SL for 2 years; Han-HA = Han migrants at HA for 1 or 5 years; Tibetan-HA = Tibetan natives at HA. The horizontal dotted and solid lines within the box represent the mean and median, respectively. *P < 0.05 *vs*. the SL counterpart; ^**†**^*P* < 0.05, Tibetans *vs*. Han Chinese at same altitude; ^#^*P* < 0.05 *vs*. females.

Race and sex differences were examined using un-paired *t* tests. Differences among all 4 Han groups were assessed using one-way analysis of variance (ANOVA), with the LSD test used for post-hoc pairwise comparisons on the parameters in women and men (Table 1), or on CBF in bilateral ICAs and VAs (Fig. 2A), separately. This helped to assess the effects of short- and long-term HA exposures on the Han lowlanders. In addition, a 2-way ANOVA was performed in all Han groups to examine the sex effects on the responses to HA, as reflected by the interaction of 4(different altitude exposure) × 2(Sex); and a 2-way ANOVA with repeated-measures general linear model (GLM) was used to assess the regional differences of CBF response to short-term HA exposure, as reflected by the interaction of 2(acute altitude exposure, i.e. SL and HA-2d) × 2(Region).

Since Han-HA-1yr and Han-HA-5yr exhibited high similarity in most of their parameters, they were combined into a larger group (i.e. Han-HA) to represent Han migrants with years of long-term adaptation to HA. This group and the other 3 long-term groups (Han-SL, Tibetan-SL and Tibetan-HA) were compared on their parameters between different altitude, race or sex using un-paired *t* tests. We also performed a 3-way ANOVA, i.e. 2(Altitude) × 2(Race) × 2(Sex), to examine interaction effects (see major results in Table 3). A 2-way ANOVA with repeated-measures GLM was further performed in Tibetan SL and HA groups to examine the regional difference of CBF response to long-term HA exposure, as reflected by the interaction of 2(Altitude) × 2(Region).

All data are presented as mean ± SD (unless otherwise stated). The relative difference between two compared groups is expressed as the percentage change (%) of the mean value relative to the reference group. A 2-tailed *P* < 0.05 was used to establish the statistical significance. Statistical analysis was performed with the SPSS 19.0 software (IBM, Armonk, NY, USA).

## Additional Information

**How to cite this article**: Liu, J. *et al*. Effects of race and sex on cerebral hemodynamics, oxygen delivery and blood flow distribution in response to high altitude. *Sci. Rep*. **6**, 30500; doi: 10.1038/srep30500 (2016).

## Figures and Tables

**Table 1:** Demographic characteristics, resting physiological values of Han groups with different HA exposures.

Variables^§^		SL (400 m)		HA (3658 m)					
		Han-SL		Han-HA-2d		Han-HA-1yr		Han-HA-5yr	
*Demographics*		*Female*	*Male*	*Female*	*Male*	*Female*	*Male*	*Female*	*Male*
Sample size, *n*		24	24	20	21	22	40	20	20
Age, years		21 ± 1	21 ± 1	19 ± 2*	19 ± 1*	20 ± 2^△^	20 ± 2^△^	23 ± 1*	23 ± 2*
Height, cm		161 ± 4	172 ± 5^#^	162 ± 3	171 ± 4^#^	162 ± 5	171 ± 6^#^	162 ± 6	173 ± 5^#^
Weight, kg		53 ± 7	63 ± 7^#^	54 ± 7	61 ± 7^#^	53 ± 7	61 ± 7^#^	53 ± 7	61 ± 6^#^
BMI, kg/m^2^		20.3 ± 2.3	21.3 ± 2.3	20.5 ± 2.2	20.9 ± 2.1	20.1 ± 1.9	20.9 ± 1.8	20.3 ± 2.3	20.5 ± 1.7
BSA, m^2^		1.54 ± 0.11	1.74 ± 0.11^#^	1.56 ± 0.11	1.71 ± 0.11^#^	1.54 ± 0.11	1.70 ± 0.12^#^	1.55 ± 0.12	1.72 ± 0.10^#^
*Systemic measurements*									
SBP, mmHg		107 ± 6	117 ± 8^#^	116 ± 7*	124 ± 10^#^*	115 ± 8*	124 ± 11^#^*	118 ± 11*	128 ± 7^#^*
DBP, mmHg		66 ± 6	66 ± 5	76 ± 9*	75 ± 11*	71 ± 9^△^	72 ± 9*^△^	78 ± 11*	78 ± 6*
MAP, mmHg		80 ± 5	83 ± 6	90 ± 8*	92 ± 10*	86 ± 8*^△^	89 ± 9*^△^	91 ± 10*	95 ± 6*
Heart rate, beats/min		69 ± 10	66 ± 8	94 ± 11*	85 ± 13^#^*	79 ± 13*	69 ± 13^#^	76 ± 13	71 ± 10
Hct, %		39.9 ± 3.2	45.7 ± 2.9^#^	42.6 ± 2.0*	48.6 ± 2.4^#^*	45.2 ± 2.2*^△^	51.4 ± 2.6^#^*	47.2 ± 4.4*	52.2 ± 3.3^#^*
Hb, gm/dL		13.5 ± 1.3	15.9 ± 1.1^#^	13.5 ± 0.9	16.0 ± 0.7^#^	15.6 ± 1.0*^△^	18.4 ± 0.9^#^*	16.5 ± 1.7*	18.9 ± 1.2^#^*
SaO_2_, %		98.2 ± 0.9	98.0 ± 0.6	89.1 ± 4.7*	87.9 ± 2.6*	92.5 ± 1.5*	92.0 ± 1.6*	92.9 ± 3.4*	92.1 ± 2.0*
CaO_2_, ml O_2_/dL		18.0 ± 1.8	21.2 ± 1.4^#^	16.3 ± 1.5*	19.1 ± 1.0^#^*	19.6 ± 1.2*^△^	23.0 ± 1.1^#^*	20.8 ± 1.8*	23.6 ± 1.6^#^*
*ICA measurements*									
Diameter, mm	*R*	4.24 ± 0.39	4.53 ± 0.30^#^	4.82 ± 0.53*	5.15 ± 0.47^#^*	4.68 ± 0.36*	4.93 ± 0.45^#^*	4.76 ± 0.48*	4.99 ± 0.51*
	*L*	4.19 ± 0.32	4.48 ± 0.22^#^	4.86 ± 0.67*	5.08 ± 0.30*	4.51 ± 0.35*	4.84 ± 0.54^#^*	4.69 ± 0.43*	4.86 ± 0.48*
CBFV, cm/s	*R*	26.6 ± 7.1	24.0 ± 3.6	24.4 ± 4.3	22.5 ± 4.3	23.6 ± 4.9	20.8 ± 3.6^#^*	21.8 ± 4.2*	19.9 ± 4.0*
	*L*	29.6 ± 5.5	25.0 ± 4.5^#^	24.2 ± 7.5*	21.8 ± 4.5*	23.2 ± 4.9*	20.9 ± 3.4^#^*	22.6 ± 4.0*	21.1 ± 4.3*
CBF, mL/min	*R*	223 ± 51	233 ± 43	269 ± 70*	281 ± 62*	241 ± 45	238 ± 54	230 ± 47	232 ± 52
	*L*	242 ± 40	236 ± 43	264 ± 80	265 ± 57	222 ± 48	233 ± 63	233 ± 48	232 ± 43
*VA measurements*									
Diameter, mm	*R*	3.00 ± 0.53	3.13 ± 0.41	3.46 ± 0.40*	3.56 ± 0.46*	3.28 ± 0.44	3.33 ± 0.34	3.33 ± 0.50*	3.38 ± 0.44*
	*L*	3.14 ± 0.44	3.21 ± 0.38	3.57 ± 0.57*	3.62 ± 0.56*	3.35 ± 0.35	3.48 ± 0.45*	3.52 ± 0.48*	3.57 ± 0.38*
CBFV, cm/s	*R*	18.7 ± 3.7	16.1 ± 3.2^#^	17.2 ± 3.5	15.0 ± 3.5	15.3 ± 2.6*	13.7 ± 2.8^#^*	14.4 ± 2.6*	13.6 ± 2.8*
	*L*	19.3 ± 4.9	17.3 ± 3.1	17.4 ± 4.9	15.1 ± 2.9*	17.7 ± 2.5^△^	15.2 ± 2.9^#^*	15.1 ± 3.2*	14.8 ± 2.3*
CBF, mL/min	*R*	81 ± 29	77 ± 29	98 ± 29	93 ± 37	80 ± 25	73 ± 21	77 ± 28	76 ± 30
	*L*	93 ± 38	86 ± 28	108 ± 49	98 ± 37	95 ± 28	88 ± 28	91 ± 37	91 ± 27
*Global brain perfusion*									
TCBF, mL/min		639 ± 84	632 ± 89	739 ± 148*	736 ± 132*	638 ± 96	632 ± 114	632 ± 89	631 ± 99
TCVR, 10^-3^mmHg∙min/mL		127 ± 19	134 ± 22	125 ± 25	128 ± 24	138 ± 23	146 ± 31	149 ± 35*	154 ± 29*
TCOD, mL O_2_/min		115 ± 20	134 ± 20^#^	120 ± 18	140 ± 23^#^	125 ± 20	145 ± 24^#^	132 ± 26*	149 ± 24^#^*
*CBF distribution*									
Q_VA_/TCBF, %		27.3 ± 5.8	25.8 ± 3.7	27.9 ± 6.4	25.9 ± 3.3	27.6 ± 4.3	25.8 ± 4.3	26.6 ± 5.0	26.6 ± 3.6

^§^Values are mean ± SD.

^#^*P* < 0.05 *vs*. females using un-paired *t* test.

*P < 0.05 *vs*. the SL counterpart (Han-SL).

^△^*P* < 0.05, Han-HA-1yr *vs*. Han-HA-5yr using one-way ANOVA for 4 Han groups. SL = sea level; HA = high altitude; Han-SL = Han natives at SL; Han-HA-2 d = Han newcomers at HA for only 2 days; Han-HA-1yr = Han migrants at HA for 1 year; Han-HA-5yr = Han migrants at HA for 5 years; BMI = body mass index; BSA = body surface area = 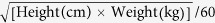; SBP = systolic blood pressure; DBP = diastolic blood pressure; MAP = mean arterial pressure; Hct = hematocrit; Hb = hemoglobin concentration; SaO_2_ = arterial blood oxygen saturation; CaO_2_ = arterial oxygen content; ICA = internal carotid artery; CBFV = cerebral blood flow velocity, i.e. the space- and time-averaged mean velocity; CBF = cerebral blood flow; VA = vertebral artery; TCBF = total CBF; TCVR = total cerebrovascular resistance; TCOD = total cerebral oxygen delivery; Q_VA_/TCBF = the percentage of bilateral VA CBFs to TCBF.

**Table 2:** Demographic characteristics, resting physiological values of Tibetan and Han groups living at SL and HA for more than one year.

Variables^§^		SL (400 m)				HA (3658 m)			
		Han-SL		Tibetan-SL		Han-HA		Tibetan-HA	
*Demographics*		*Female*	*Male*	*Female*	*Male*	*Female*	*Male*	*Female*	*Male*
Sample size, *n*		24	24	26	20	42	60	37	38
Age, years		21 ± 1	21 ± 1	21 ± 1	21 ± 1	22 ± 2	21 ± 3	21 ± 1^†^	21 ± 3
Height, cm		161 ± 4	172 ± 5^#^	161 ± 4	172 ± 5^#^	162 ± 6	172 ± 6^#^	161 ± 6	170 ± 6^#^
Weight, kg		53 ± 7	63 ± 7^#^	51 ± 4	62 ± 7^#^	53 ± 7	61 ± 7^#^	52 ± 6	60 ± 8^#^
BMI, kg/m^2^		20.3 ± 2.3	21.3 ± 2.3	19.8 ± 1.9	20.8 ± 2.1	20.2 ± 2.1	20.8 ± 1.8	20.2 ± 1.7	20.6 ± 2.4
BSA, m^2^		1.54 ± 0.11	1.74 ± 0.11^#^	1.52 ± 0.07	1.72 ± 0.11^#^	1.54 ± 0.12	1.71 ± 0.10^#^	1.53 ± 0.10	1.68 ± 0.12^#^
*Systemic measurements*									
SBP, mmHg		107 ± 6	117 ± 8^#^	114 ± 9^†^	123 ± 11^#†^	117 ± 10*	125 ± 10^#^*	112 ± 9^†^	121 ± 10^#^
DBP, mmHg		66 ± 6	66 ± 5	67 ± 6	67 ± 7	74 ± 10*	74 ± 9*	68 ± 7^†^	69 ± 10^†^
MAP, mmHg		80 ± 5	83 ± 6	83 ± 7	86 ± 8	88 ± 9*	91 ± 9*	83 ± 7^†^	87 ± 9^#†^
Heart rate, beats/min		69 ± 10	66 ± 8	72 ± 11	69 ± 12	77 ± 13*	70 ± 12^#^	70 ± 7^†^	70 ± 11
Hct, %		39.9 ± 3.2	45.7 ± 2.9^#^	34.9 ± 3.5^†^	40.3 ± 4.4^#†^	46.1 ± 3.6*	51.6 ± 2.8^#^*	42.0 ± 4.7*^†^	47.8 ± 3.6^#^*^†^
Hb, gm/dL		13.5 ± 1.3	15.9 ± 1.1^#^	11.2 ± 1.3^†^	13.2 ± 1.5^#†^	16.0 ± 1.4*	18.6 ± 1.0^#^*	14.4 ± 1.8*^†^	16.8 ± 1.3^#^*^†^
SaO_2_, %		98.2 ± 0.9	98.0 ± 0.6	97.9 ± 0.5	97.9 ± 0.4	92.7 ± 2.5*	92.0 ± 1.7*	92.7 ± 2.1*	92.1 ± 2.1*
CaO_2_, ml O_2_/dL		18.0 ± 1.8	21.2 ± 1.4^#^	14.9 ± 1.7^†^	17.6 ± 2.0^#†^	20.2 ± 1.6*	23.2 ± 1.3^#^*	18.2 ± 2.2*^†^	21.0 ± 1.5^#^*^†^
*ICA measurements*									
Diameter, mm	*R*	4.24 ± 0.39	4.53 ± 0.30^#^	4.27 ± 0.40	4.55 ± 0.30^#^	4.72 ± 0.42*	4.95 ± 0.47^#^*	4.41 ± 0.51^†^	4.60 ± 0.44^†^
	*L*	4.19 ± 0.32	4.48 ± 0.22^#^	4.32 ± 0.33	4.51 ± 0.34	4.60 ± 0.39*	4.85 ± 0.51^#^*	4.36 ± 0.49^†^	4.63 ± 0.47^#†^
CBFV, cm/s	*R*	26.6 ± 7.1	24.0 ± 3.6	27.7 ± 4.6	27.6 ± 4.4^†^	22.7 ± 4.6*	20.5 ± 3.8^#^*	23.4 ± 5.4*	20.8 ± 5.4^#^*
	*L*	29.6 ± 5.5	25.0 ± 4.5^#^	29.1 ± 3.5	25.8 ± 4.3^#^	22.9 ± 4.4*	21.0 ± 3.7^#^*	21.8 ± 6.8*	19.9 ± 5.1*
CBF, mL/min	*R*	223 ± 51	233 ± 43	238 ± 50	270 ± 51^#†^	236 ± 46	236 ± 53	215 ± 65	207 ± 62*^†^
	*L*	242 ± 40	236 ± 43	255 ± 42	249 ± 56	227 ± 48	233 ± 57	194 ± 59*^†^	201 ± 58*^†^
*VA measurements*									
Diameter, mm	*R*	3.00 ± 0.53	3.13 ± 0.41	2.97 ± 0.47	3.06 ± 0.41	3.30 ± 0.46*	3.35 ± 0.37*	3.11 ± 0.39^†^	3.27 ± 0.52
	*L*	3.14 ± 0.44	3.21 ± 0.38	3.07 ± 0.41	3.12 ± 0.42	3.43 ± 0.42*	3.51 ± 0.42*	3.27 ± 0.33*	3.34 ± 0.56
CBFV, cm/s	*R*	18.7 ± 3.7	16.1 ± 3.2^#^	20.2 ± 3.8	17.9 ± 3.7^#^	14.9 ± 2.6*	13.7 ± 2.8^#^*	14.7 ± 3.9*	13.4 ± 3.2*
	*L*	19.3 ± 4.9	17.3 ± 3.1	18.7 ± 3.3	16.0 ± 2.9^#^	16.4 ± 3.1*	15.1 ± 2.7^#^*	15.6 ± 3.5*	14.1 ± 2.7^#^*
CBF, mL/min	*R*	81 ± 29	77 ± 29	88 ± 37	82 ± 30	79 ± 26	74 ± 24	69 ± 26*	71 ± 30
	*L*	93 ± 38	86 ± 28	86 ± 30	77 ± 33	93 ± 32	89 ± 27	79 ± 21^†^	78 ± 31
*Global brain perfusion*									
TCBF, mL/min		639 ± 84	632 ± 89	668 ± 92	677 ± 112	635 ± 92	631 ± 109	556 ± 105*^†^	557 ± 121*^†^
TCVR, 10^−3^ mmHg∙min/mL		127 ± 19	134 ± 22	126 ± 20	132 ± 38	143 ± 29*	149 ± 30*	154 ± 34*	162 ± 35*
TCOD, mL O_2_/min		115 ± 20	134 ± 20^#^	100 ± 17^†^	119 ± 23^#†^	128 ± 23*	146 ± 24^#^*	101 ± 20^†^	116 ± 24^#†^
*CBF distribution*									
Q_VA_/TCBF, %		27.3 ± 5.8	25.8 ± 3.7	26.1 ± 5.0	23.3 ± 3.4^#†^	27.1 ± 4.6	26.1 ± 4.1	27.3 ± 6.4	27.2 ± 5.4*

^§^Values are mean ± SD.

^#^*P* < 0.05 *vs*. females,

^†^*P* < 0.05 *vs*. Han group, and

^*^P < 0.05 *vs*. the SL counterpart, using un-paired *t* test. SL = sea level; HA = high altitude; Han-SL = Han natives at SL; Tibetan-SL = Tibetan migrants staying at SL for 2 years; Han-HA = Han migrants at HA for 1 or 5 years; Tibetan-HA = Tibetan natives at HA; BMI = body mass index; BSA = body surface area = 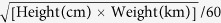 ; SBP = systolic blood pressure; DBP = diastolic blood pressure; MAP = mean arterial pressure; Hct = hematocrit; Hb = hemoglobin concentration; SaO_2_ = arterial blood oxygen saturation; CaO_2_ = arterial oxygen content; ICA = internal carotid artery; CBFV = cerebral blood flow velocity, i.e. the space- and time-averaged mean velocity; CBF = cerebral blood flow; VA = vertebral artery; TCBF = total CBF; TCVR = total cerebrovascular resistance; TCOD = total cerebral oxygen delivery; Q_VA_/TCBF = the percentage of bilateral VA CBFs to TCBF.

**Table 3:** Race-, sex-, and altitude-related differences of major parameters among young healthy Tibetans and Han Chinese examined by 3-way ANOVA^*^.

	Main effect			2-Way interaction		
	Altitude	Race	Sex	Altitude × Race	Altitude × Sex	Race × Sex
DF	1	1	1	1	1	1
***MAP***						
F value	**19.213**	1.180	**10.000**	**16.490**	0.038	0.115
*P* value	**<0.001**	0.278	**0.002**	**<0.001**	0.845	0.735
***Hct***						
F value	**211.274**	**98.893**	**147.974**	1.706	0.002	0.015
*P* value	**<0.001**	**<0.001**	**<0.001**	0.193	0.967	0.901
***CaO***_***2***_						
F value	**159.637**	**168.345**	**190.278**	**8.588**	0.001	0.855
*P* value	**<0.001**	**<0.001**	**<0.001**	**0.004**	0.981	0.356
***TCBF***						
F value	**20.019**	2.294	<0.001	**18.276**	0.009	0.145
*P* value	**<0.001**	0.131	0.990	**<0.001**	0.926	0.704
***TCVR***						
F value	**32.488**	2.036	2.855	2.869	0.002	0.004
*P* value	**<0.001**	0.155	0.092	0.091	0.966	0.948
***TCOD***						
F value	**4.399**	**62.359**	**40.084**	**5.417**	0.134	0.027
*P* value	**0.037**	**<0.001**	**<0.001**	**0.021**	0.715	0.869
***Q***_***VA***_***/TCBF***						
F value	**4.251**	0.961	**4.694**	**4.103**	1.563	0.021
*P* value	**0.040**	0.328	**0.031**	**0.044**	0.212	0.885

^*^Sample size, n = 271. Altitude, Race and Sex are all dichotomous, with dummy codes as 0 and 1 for sea-level and high-altitude, 0 and 1 for Han Chinese and Tibetans, and 0 and 1 for females and males, respectively. The significant (*P* < 0.05) results are highlighted in bold. Results on 3-way interaction are not presented because none of them are significant. The homogeneity of variance between each cell was confirmed using Levene’s test (*P* > 0.05). DF = degree of freedom; MAP = mean arterial pressure; Hct = hematocrit; CaO_2_ = arterial oxygen content; TCBF = total cerebral blood flow; TCVR = total cerebrovascular resistance = MAP/TCBF; TCOD = total cerebral oxygen delivery; QVA/TCBF = the percentage of posterior CBF to TCBF.
